# Identifying Patterns of Similarities and Differences between Gesture Production and Comprehension in Autism and Typical Development

**DOI:** 10.1007/s10919-021-00394-y

**Published:** 2022-01-06

**Authors:** Nevena Dimitrova, Şeyda Özçalışkan

**Affiliations:** 1 Faculty of Social Work (HETSL|HES-SO), University of Applied Sciences and Arts Western Switzerland, 14 chemin des Abeilles, 1010 Lausanne, Switzerland; 2 Department of Psychology, Georgia State University, P.O. Box 5010, Atlanta, GA 30302 USA

**Keywords:** Nonverbal communication, Gesture production, Gesture comprehension, Developmental trajectory, Autism

## Abstract

Production and comprehension of gesture emerge early and are key to subsequent language development in typical development. Compared to typically developing (TD) children, children with autism spectrum disorders (ASD) exhibit difficulties and/or differences in gesture production. However, we do not yet know if gesture production either shows similar patterns to gesture comprehension across different ages and learners, or alternatively, lags behind gesture comprehension, thus mimicking a pattern akin to speech comprehension and production. In this study, we focus on the gestures produced and comprehended by a group of young TD children and children with ASD—comparable in language ability—with the goal to identify whether gesture production and comprehension follow similar patterns between ages and between learners. We elicited production of gesture in a semi-structured parent–child play and comprehension of gesture in a structured experimenter-child play across two studies. We tested whether young TD children (ages 2–4) follow a similar trajectory in their production and comprehension of gesture (Study 1) across ages, and if so, whether this alignment remains similar for verbal children with ASD (*M*_age_ = 5 years), comparable to TD children in language ability (Study 2). Our results provided evidence for similarities between gesture production and comprehension across ages and across learners, suggesting that comprehension and production of gesture form a largely integrated system of communication.

## Introduction

Children produce and understand gesture at an early age (Bates, 1976; Greenfield & Smith, 1976; Hodges et al., 2018; Iverson et al., 1994). These early gestures precede and predict upcoming changes in children’s spoken language development both in typical development (Iverson & Goldin-Meadow, 2005; Özçalışkan & Goldin-Meadow, 2005a) and in autism (Gulsrud et al., 2014; Mastrogiuseppe et al., 2015; Özçalışkan et al., 2016, 2017). More important, evidence of delays and/or differences in speech is observable first in gesture, highlighting gesture as an important early diagnostic tool to detect the timing and the extent of delays in spoken language development, particularly for children with autism spectrum disorders (ASD; Özçalışkan et al., 2016; Ramos-Cabo et al., 2019).

Earlier research on TD children focused on either production or comprehension of gesture, leaving the link between the two unexamined. The scarcity of research becomes even more pronounced for children with developmental disorders, such as ASD. As such, we do not yet know whether production and comprehension of gesture show similar trajectories of change in typical development, and if so, how these patterns compare to children with ASD, who show difficulties in gesture production (Colgan et al., 2006; Mitchell et al., 2006; Mundy et al., 1986). One possibility is that children with ASD show the same difficulty with gesture comprehension as they do with gesture production, suggesting that the two processes are coupled in communicative development. Another possibility, however, is that, unlike production, children with ASD might show strengths in gesture comprehension, raising the possibility of distinct processes associated with each communicative ability.

In this study, we investigate similarities and differences between patterns of gesture production and comprehension in TD children and language-comparable children with ASD across two studies. Our goal is to identify whether the production and comprehension of gesture follow a similar developmental trajectory between ages 2–4 in typical development (Study 1), and whether the overall patterns of production and comprehension observed in TD children also extend to children with ASD (Study 2). Identifying similarities (or their lack) in patterns of gesture production and comprehension across different ages (2, 3, 4) and learners (TD, ASD) serves as an important first step in providing a more comprehensive framework in understanding early communicative development, and consequently in devising more effective instructional strategies for better learning outcomes in both nonverbal and verbal communicative development at the early ages.

## Study 1

### Production and Comprehension of Gesture in TD Children

Children start producing (Bates, 1976; Bates et al., 1979; see Capone & McGregor, 2004 for a review) and comprehending (Camaioni et al., 2004; Colonnesi et al., 2008; Morford & Goldin-Meadow, 1992) gestures somewhere between 10–12 months of age, several months before they start producing words. Earlier work focusing on either production or comprehension of gesture suggests a similar timeline in the production of different *gesture types*. For example, children begin to produce deictic gestures that indicate objects (e.g., point at balloon) around age 1 (Colonnesi et al., 2010; Özçalışkan & Goldin-Meadow, 2005b); they also show the ability to comprehend deictic gestures around the same age, by successfully following an adult’s pointing gesture that indicates a referent (Carpenter et al., 1998; Liszkowski et al., 2006; Scaife & Bruner, 1975). Children that produce deictic gestures also show better comprehension of such gestures (Behne et al., 2012; Woodward & Guajardo, 2002), further marking a close association between comprehension and production of deictic gestures.

The temporal association between comprehension and production also becomes evident in other gesture types. Children begin to produce iconic gestures that characterize referents (e.g., flapping palms for flying) along with conventional gestures that express culturally-prescribed meanings with frozen iconic gesture forms (e.g., waving palm for goodbye) around 2 years (Iverson et al., 1994; Özçalışkan & Goldin-Meadow, 2011; Özçalışkan et al., 2014). It is around the same age that we also observe an increase in children’s comprehension of iconic gestures (Hodges et al., 2018; Namy et al., 2004; Stanfield et al., 2014): Compared to one-and-a-half-year-old children who equally associate both an iconic and an arbitrary gesture to a referent, 2-year-old children show greater preference to associate an iconic gesture with a referent, showing greater sensitivity to iconicity (Namy et al., 2004). This preference also becomes evident when children are asked to identify the referent of a novel iconic gesture: following observation of an action performed on a novel object, 2-year-old children were more likely to associate the iconic gesture that depicts the same action than 1-year-old children (Namy, 2008). Children’s relatively later grasp of iconic gestures has also been shown by Shore et al. (1990) who examined both production and comprehension of iconic gestures in the same group of children. Two-and-a half-year-old children were asked to identify a referent from a set of three objects (cup, shoe, brush) based on information provided by an iconic gesture (e.g., pantomime of drinking). Half the children mimicked the gestures of the experimenter without explicit instruction to do so, and performed better in the comprehension task by identifying the correct referent for the gesture more frequently than children who did not mimic the experimenter’s gestures. Overall, previous research suggests that children’s production and comprehension of different gesture types follows a similar trajectory in development, from deictic to iconic gestures.

Shortly after producing their first words, children begin to combine words with gestures, first expressing the same information as speech (reinforcing gesture + speech; “balloon” + point at balloon), then adding further information to speech (supplementary gesture + speech; e.g., “mine” + point at balloon; Butcher & Goldin-Meadow, 2000; Greenfield & Smith, 1976; Özçalışkan & Goldin-Meadow, 2005a, 2005b). The developmental trajectory observed in the production of gesture + speech combinations—from reinforcing to supplementary—also becomes evident in the comprehension of gesture + speech. An earlier study (Morford & Goldin-Meadow, 1992) examined 1–2 -year-old children’s comprehension of conventional and pointing gestures with speech and showed that children showed earlier comprehension of gestures that reinforce speech than gestures that supplement speech. Additionally, the type of the gesture in a gesture–speech combination mattered: at 1;3, children could act on an object that was uniquely identified in a deictic gesture + speech combination (“open” + point at bag), and at 1;8 they could do so when presented with a conventional gesture + speech combination (“ball” + give gesture; Morford & Goldin-Meadow, 1992). The effect of gesture type in multi-modal communications was observable for the production and comprehension of iconic gesture + speech combinations as well. Children begin to produce iconic gesture + speech combinations in their communications between ages 2 and 3—first to convey the same information as speech, and only later to convey different information than speech (Özçalışkan & Goldin-Meadow, 2005a, 2009, 2011; Özçalışkan et al., 2014). Similarly, earlier research that examined children’s comprehension of iconic gesture + speech combinations—with gesture adding either action and/or feature information also marks comprehension abilities between ages 2–3 (Hodges et al., 2018; Stanfield et al., 2014). Results suggest that production and comprehension of gesture + speech types also follow a similar trajectory in development, from reinforcing to supplementary gesture + speech combinations, accompanied first with deictic then with conventional and iconic gestures.

### The Current Study

Earlier work focused on production *or* comprehension of gesture in young children, leaving the parallel changes in production and comprehension of gesture in the same group of children mostly unexamined. The one study (Shore et al., 1990) that examined comprehension and production with the same sample focused only on iconic gestures that are produced without speech, limiting the generalizability of the results to other types of gestures. Thus, we do not yet know whether developmental changes in children’s production and comprehension of different gestures and gesture + speech combinations follow a similar trajectory, or whether gesture production lags behind gesture comprehension—following a pattern in speech development (e.g., Bates et al., 1989; Bornstein & Hendricks, 2012). We examined this question by studying patterns of gesture production and comprehension in a sample of 2–4 -year-old TD children. The patterns of gesture comprehension in these children were reported in earlier work (Dimitrova et al., 2017), which showed better comprehension of deictic gestures and reinforcing gesture + speech combination than iconic gestures and supplementary gesture + speech combinations. In this study, we extended on these findings by including the same children’s production of gesture—which was not reported in earlier work, along with their comprehension to determine whether the two processes of gestural communication would follow similar trajectories. More specifically, we asked whether 2–4 -year-old children would show a similar developmental trajectory in their production and comprehension of different types of gestures and gesture + speech combinations. We predicted that children’s production and comprehension would show a similar developmental timeline, with greater production and better comprehension of deictic gestures and reinforcing gesture + speech combinations than iconic gestures and supplementary gesture + speech combinations at the younger ages. Our prediction was based on studies that independently examined either production (e.g., Iverson et al., 1994; Özçalışkan & Goldin-Meadow, 2005a, 2009) or comprehension of gesture (e.g., Morford & Goldin-Meadow, 1992; Stanfield et al., 2014).

## Method

### Participants

The sample consisted of 41 children, including 13 2-year-olds (*M*_age_ = 2;71, range 2;2–2; 11, 4 males), 15 3-year-olds (*M*_age_ = 3;5, range = 3;00–3;11, 8 males), and 13 4-year-olds (*M*_age_ = 4;6, range = 4;1–5;0, 5 males).^1^ As part of our inclusion criteria, all TD children were between ages 2-to-5, had no known cognitive or language disorders based on parental report, were learning English as their native language, and scored within the typical range in Social Responsiveness Scale (SRS, Constantino & Gruber, 2005; *M* = 43.66; *SD* = 4.75, range = 34–56). The children were Caucasian (58%), African-American (32%), or of mixed race (10%). Most parents had either a college (49%) or a postgraduate degree (34%); they all provided written consent for their child’s participation prior to the study.

### Procedure for Data Collection

Each child was tested individually. They first completed two standardized tests, assessing receptive (Picture Vocabulary Test, PPVT-IV; Dunn & Dunn, 2007) and expressive (Expressive Language Test, EVT-2; Williams, 2007) vocabulary—using the most up-to-date versions of the two tests at the time of data collection. Afterwards they completed two tasks, one assessing their production and one testing their comprehension of gesture. All responses were video-recorded. The research protocol was approved by the ethics board of a large research university in Southeastern United States.

**Fig. 1:**
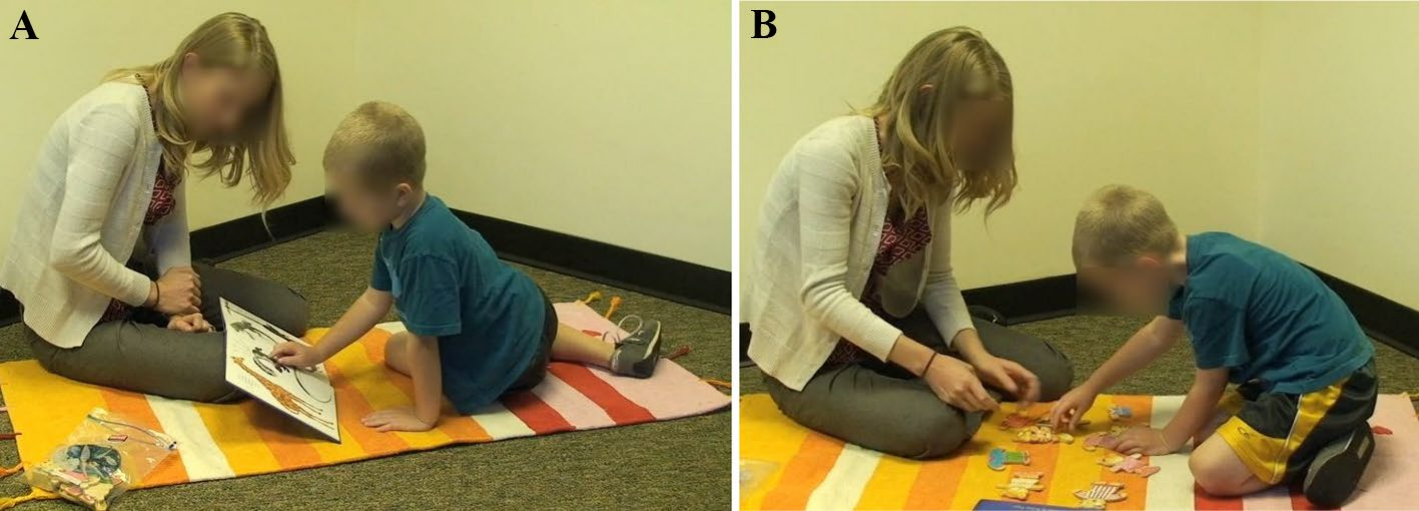
Snapshots from child-parent play with picture book (**A**) and puzzle (**B**)

*Gesture production* Children’s production of gesture was assessed during a semi-structured play between parent and child in a laboratory. Each dyad was provided with two props by the experimenter (a picture book, a puzzle)—both known to elicit gestures in earlier work (Baumann et al., 2019). The parents were told to play with their children as naturally as they would in their everyday lives—without any explicit instruction to gesture or respond to gesture—using each prop for five minutes, resulting in 10 min of gesture production per child (see Fig. 1 for screenshots from the gesture production task involving parent–child play).

*Gesture comprehension* After completion of the gesture production task, each child proceeded with the gesture comprehension task. The child was asked to sit at a table, right across from a female experimenter, and the parent was asked to sit in an armchair behind the child outside the child’s visual field. The parent was also asked to remain quiet during the administration of the gesture comprehension task by the experimenter. The experimenter was blind to the hypotheses of the study.

**Fig. 2:**
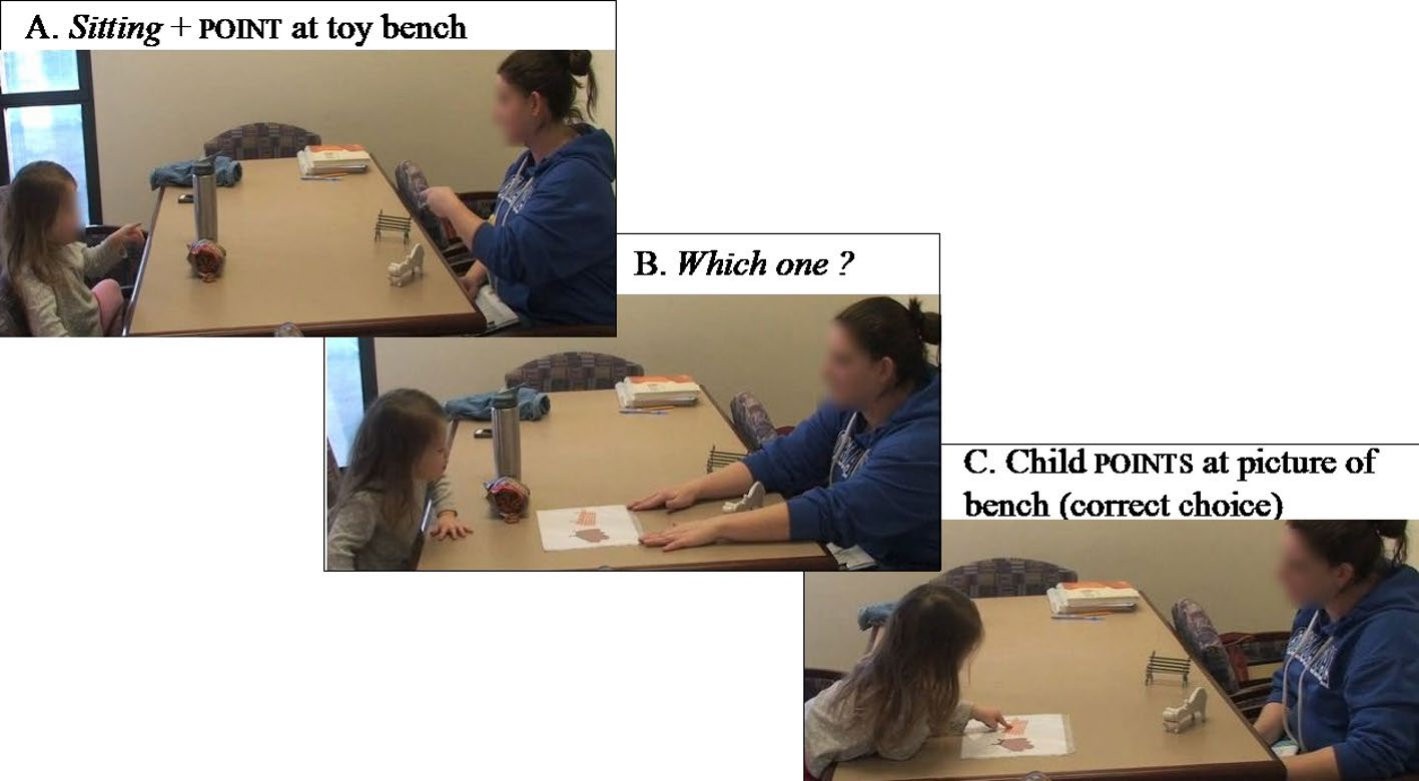
Snapshots from gesture comprehension task testing children’s comprehension of a supplementary gesture + speech combination (**A**. Sitting + point at toy bench), followed by a forced-choice question by the experimenter (**B**. Which one?), and a response by the child (**C**. child points at picture of bench)

The gesture comprehension task assessed children’s comprehension of 3 different gesture types (deictic, conventional, iconic) across 3 different communicative modalities with gesture (i.e., gesture only, reinforcing gesture–speech combination, supplementary gesture–speech combination) and one without gesture (speech only), resulting in 36 test items (3 gesture types × 4 communicative modalities; see Dimitrova et al., 2017 for details). In this study, given our focus, we only included children’s responses to the 3 communicative modalities with gesture (i.e., gesture only, reinforcing gesture + speech, supplementary gesture + speech; see Fig. 2 for snapshots from the comprehension task).

### Procedure for Data Transcription and Coding

*Gesture production* All child responses were transcribed for speech and later coded for gesture. We treated sounds that referred to entities, properties, or events (e.g., ‘doggie’, ‘open’), along with onomatopoeic (e.g., ‘meow’), and conventionalized evaluative sounds (e.g., ‘oopsie’) as words, following earlier work (Özçalışkan & Goldin-Meadow, 2005b). We segmented speech into utterances, using grammatical structure, pauses, and intonation as markers, following Hoff (2012).

We further coded all video records for gesture. We defined gesture as a symbolic communicative hand movement that did not involve direct manipulation of objects (e.g., twisting a jar open, giving an object to the interlocutor). The only exception was the ‘hold-up’ gesture in which the child showed an object in hand to share attention about the object, without offering the object to the parent. These ‘hold-up’ gestures served the same function as pointing gestures by bringing attention to the object and were also coded as gestures, following earlier work (Butcher & Goldin-Meadow, 2000; Iverson et al., 1999; Özçalışkan & Goldin-Meadow, 2005a). Each gesture was further coded into types based on its form and the informational relation it held to the accompanying speech (i.e., gesture + speech; see Table 1 for definitions and examples). The majority of the gestures were produced with speech (63%); a few were also produced without speech (37%), both were included in the gesture type analysis.

**Table 1:** Types of gestures and gesture + speech combinations

Gesture types	Definition	Example
Deictic	Identify referents by pointing with a finger or palm (deictic point) or by holding them up next the gesturer’s body (deictic show) with the goal to share information about the referent	Point at cat to identify cat; Hold up bottle to identify bottle
Conventional	Use hand or body in culturally-prescribed ways to convey shared conventional meanings	Nod head to convey affirmation; Flip palms to convey lack of knowledge
Iconic	Use hand or body to symbolically represent an entity by characteristic action or feature	Flap arms to convey bird flying; Form a circle with cupped palms to convey round ball
*Gesture* + *speech combinations*		
Reinforcing	Gesture conveys the same information as the accompanying speech	“Bike” + point at bike to identify bike; “Throw” + move empty fist forcefully forward to convey throwing
Supplementary	Gesture adds semantic information not found in the accompanying speech	“Play” + point at ball to identify ball; “Mommy” + place fisted palm next to ear to convey telephone

Reliability for gesture coding was assessed with two coders blind to the study hypotheses. Both coders were trained on video records that were part of a different data set until they reached 80% agreement on coding. One coder then coded the entire data and the second coder independently coded a randomly selected 15% of the data for the current study. Agreement between coders was assessed by computing percent agreement on each key measure of gesture coding. Intercoder agreement was 82% for identification of gesture, 100% for coding gesture into types (deictic, conventional, iconic) and 86% for coding gesture + speech into types (reinforcing, supplementary).

*Gesture comprehension* The child’s response to the forced-choice question in each test trial in the gesture comprehension task received a score of ‘0’ (incorrect) or ‘1’ (correct), resulting in a maximum possible score of 12 for each gesture type (12 for deictic, 12 for iconic, 12 for conventional) and a maximum possible score of 9 for each gesture + speech type (9 for reinforcing gesture + speech combinations and 9 for supplementary gesture + speech combinations). One coder scored all responses using video records. A second coder—blind to study hypotheses—scored a randomly selected 20% of the responses in each age group. The agreement between coders was 98%.

### Scoring and Analysis

We computed each child’s gesture comprehension score across all items with gesture (range = 0–36), and separately for each gesture type (range = 0–12) and gesture + speech combination type (range = 0–9). We also tallied each child’s gesture production (i.e., gesture tokens), and separately for each gesture type (deictic, conventional, iconic) and each gesture + speech combination type (reinforcing, supplementary).

We examined differences in children’s overall rates of gesture production and comprehension using one-way ANOVAs with age as a between-subjects factor. Gesture rates for both production and comprehension showed group differences (see Table 2). We, therefore, converted all raw frequencies into proportions separately for gesture production^2^ and gesture comprehension^3^ for each individual child, arcsine-transformed the proportions, and conducted all analyses on the arcsine-transformed proportions.

**Table 2:** Mean number (standard deviation in parentheses) of gesture production (upper panel) and gesture comprehension (lower panel) by 2-, 3-, and 4-year-old typically developing (TD) children

		Age 2 (*n* = 13)	Age 3 (*n* = 15)	Age 4 (*n* = 13)
		Mean (*SD*)	Mean (*SD*)	Mean (*SD*)
*PRODUCTION*				
Gesture types	Overall	19.23 (9.31)	21.60 (13.05)	29.08 (18.53)
	Deictic	16.00 (8.49)	15.67 (9.90)	21.46 (17.69)
	Conventional	2.85 (3.69)	5.40 (4.95)	7.31 (8.22)
	Iconic	0.38 (0.76)	0.53 (0.91)	0.31 (0.63)
Gesture + speech combinations	Reinforcing	2.30 (2.13)	3.93 (3.45)	6.15 (5.74)
	Supplementary	4.76 (6.12)	4.33 (8.34)	7.61 (11.29)
*COMPREHENSION*				
Gesture types	Overall	21.77 (4.51)	26.20 (4.32)	30.38 (3.70)
	Deictic	8.62 (2.46)	9.87 (2.03)	11.08 (1.18)
	Conventional	5.92 (1.60)	7.73 (1.94)	9.23 (1.30)
	Iconic	7.23 (1.69)	8.60 (2.16)	10.08 (1.84)
Gesture + speech combinations	Reinforcing	5.84 (2.03)	7.06 (1.98)	8.30 (1.18)
	Supplementary	4.61 (1.19)	5.93 (1.33)	6.46 (1.61)

We assessed differences in patterns of gesture production and comprehension with two separate mixed ANOVAs with age (2, 3, 4 years) as a between-subjects and either gesture type (deictic, conventional, iconic) or gesture + speech combination type (reinforcing, supplementary) as within-subject factors. All posthoc multiple comparisons were corrected using Bonferroni.

## Results

**Fig. 3:**
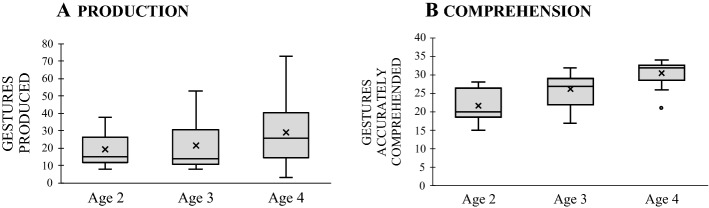
Mean number of gestures children produced (**A**) and accurately comprehended (**B**) by age. The boxplot shows both the spread and the centers of the scores separately for production and comprehension. The measure of spread includes the interquartile range from 1st to 3rd quartiles (marked by the range of shaded columns) and score range (whiskers); the measure of centers includes the mean (x) and the median (marked by the dark horizontal line within the shaded column)

We first examined developmental changes in children’s overall production and comprehension of gesture. First looking at *production*, we found that children tended to increase their production with age –a tendency that was not statistically reliable, *F*(2, 38) = 1.714, *p* = 0.189 (see Fig. 3A). Turning next to *comprehension*, we found that children improved with age, *F*(2, 38) = 13.649, *p* = 0.0001, η^2^_p_ = 0.418, showing significant increases in gesture comprehension from both age 2 to 3 (*p* = 0.025) and age 3–4 (*p* = 0.037; see Fig. 3B).

**Fig. 4:**
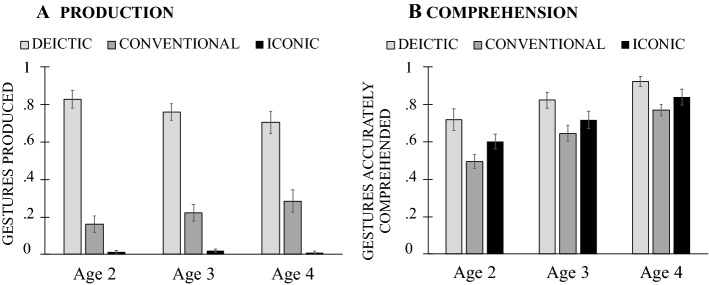
Mean proportion of deictic, conventional and iconic gestures children produced (**A**) and accurately comprehended (**B**) by age

Next, we asked whether children’s production and comprehension of different types of gestures and gesture + speech combinations showed the same developmental pattern. First looking at *gesture types*, we found that children’s *production* showed an effect of gesture type, *F*(2, 76) = 143.693, *p* = 0.0001, η^2^_p_ = 0.791, but no effect of age, *F*(2, 38) = 0.737, *p* = 0.485, η^2^_p_ = 0.037, or Gesture Type × Age interaction, *F*(4, 76) = 0.506, *p* = 0.731, η^2^_p_ = 0.070 (Fig. 4A). Children produced a greater proportion of deictic than both conventional and iconic gestures (*p*_*s*_ ≤ 0.001)—a pattern that remained unchanged over developmental time. A mostly similar pattern was also evident in children’s *comprehension* of different gesture types, with an effect of gesture type, *F*(2, 76) = 20.428, *p* = 0.001, η^2^_p_ = 0.350, an effect of age, *F*(2, 38) = 12.473, *p* = 0.0001, η^2^_p_ = 0.396, but no Gesture Type × Age interaction, *F*(4, 76) = 0.063, *p* = 0.992, η^2^_p_ = 0.003. Children increased their gesture comprehension abilities over time (*p*_*s*_ < 0.05), showing best comprehension skills for deictic gestures, followed by iconic and conventional ones (*p*_*s*_ ≤ 0.005; Fig. 4B).

**Fig. 5:**
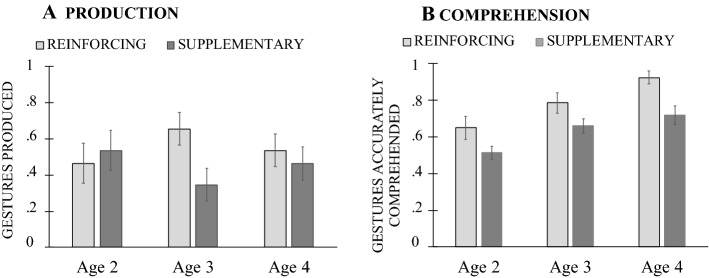
Mean proportion of reinforcing and supplementary gesture + speech combinations children produced (5A) and accurately comprehended (5B) by child age

Turning next to gesture + speech combinations, we found that children’s *production* showed no effect of combination type, *F*(1, 38) = 0.060, *p* = 0.808, η^2^_p_ = 0.002, no effect of age, *F*(2, 38) = 0.281, *p* = 0.757, η^2^_p_ = 0.015, and no interaction between Age × Combination Type, *F*(2, 38) = 0.281, *p* = 0.757, η^2^_p_ = 0.015 (see Fig. 5A). Children’s gesture *comprehension,* however, showed an effect of age, *F*(2, 38) = 9.937, *p* = 0.0001, η^2^_p_ = 0.343—with a significant difference between 2- and 4-year-olds (*p* < 0.001), an effect of gesture + speech type, *F*(1, 38) = 23.919, *p* = 0.0001, η^2^_p_ = 0.386, but no interaction, *F*(2, 38) = 1.296, *p* = 0.285, η^2^_p_ = 0.064. Overall, children showed better comprehension of reinforcing than supplementary gesture + speech combinations (*p* < 0.001; see Fig. 5B).

## Discussion

In study 1, we examined gesture production and comprehension in 2- to 4-year-old TD children in order to determine whether production and comprehension of gesture follow the same trajectory in typical development. Our results showed that children’s gesture comprehension improved by age. Children also slightly increased their gesture production at the later ages—a tendency that, however, did not reach statistical significance. Children’s production and comprehension of different gesture showed similarities however, with an advantage for deictic gestures compared to iconic gestures. Children also showed better performance for reinforcing than supplementary gesture + speech combinations—a difference that was only reliable for gesture comprehension.

Why do children improve their comprehension and tend to increase their production of gesture between ages 2–4? Gesture—both *doing* and *observing* gesture—forms an integral aspect of early communication. Gesture provides young children a venue to communicate about referents before they can do so with words (Iverson & Goldin-Meadow, 2005; Limia et al., 2019; Özçalışkan et al., 2017; Öztürk et al., 2021); and children develop larger vocabularies in gesture compared to speech (Goodwyn & Acredolo, 1998). Even after the production of words around age 1, gesture continues to offer an easy-to-use tool to convey ideas before children can express them exclusively in speech—from first sentences (Butcher & Goldin-Meadow, 2000; Iverson et al., 2008; Özçalışkan et al., 2018; Özçalışkan & Goldin Meadow, 2005a) between ages 2–3 to first narratives (Demir et al., 2015; Stites & Özçalışkan, 2017) and explanations (e.g., Özçalışkan, 2007; Özçalışkan et al., 2009) between ages 4–6 (see Goldin-Meadow, 2014; Özçalışkan & Hodges, 2016 for reviews). Producing gesture remains a robust communicative activity in young children’s communications. Children not only produce gestures on their own, but also observe their parents gesture in their interactions. Importantly, children can not only glean information from these gestures, but also use parents as models for the gestures that they themselves produce (Iverson et al., 1999; Özçalışkan & Goldin-Meadow, 2005a; see Özçalışkan & Dimitrova, 2013 for a review). Thus, both production and comprehension of gesture serve important communicative functions in laying the groundwork for speech development.

Why do children show an earlier production and comprehension of deictic than iconic gestures? One reason could be the form of deictic gestures, which does not change as a function of its referent. Children can indicate or observe others indicate an array of referents with a relatively simple hand shape (i.e., extended index finger). As such deictic gestures place a lighter cognitive load on the child, making their production or comprehension easier and earlier in development. In contrast, conventional and iconic gestures are typically more complex in form, involving either iconic resemblance and/or socially prescribed meaning. This poses a greater cognitive load in not only understanding and remembering, but also producing the gesture—a difference that results in later emergence of such gestures in both comprehension and production (e.g., Hodges et al., 2018; Özçalışkan et al., 2014; Stanfield et al., 2014). Another potential reason could be the frequency of deictic gestures in parental gesture input, compared to other gesture types. Parents adjust the gestural input to their children, producing mostly simpler deictic gestures at the early ages, compared to iconic gestures that appear later (Bekken, 1989; O’Neill et al., 2005; Özçalışkan et al., 2018; Pınar et al., 2021).

Why do children show better production and comprehension of reinforcing gesture + speech combinations at an earlier age than supplementary gesture + speech combinations? Similar to gesture types, one possible explanation could be relative ease of the two combination types. Compared to reinforcing gesture + speech, where gesture and speech express the same meaning, supplementary gesture + speech combinations impose heavier cognitive demands as the child needs to integrate different pieces of information across modalities—a challenge that might require more time in both comprehension and production. Another explanation could also be the gesture + speech input: parents of children overwhelmingly prefer reinforcing over supplementary combinations in their early communications with their children (O’Neill et al., 2005; Özçalışkan & Goldin-Meadow, 2005a; Özçalışkan et al., 2018)—a pattern that might serve as a factor in children’s own production and comprehension of the different gesture + speech combinations.

One limitation of our study was its cross-sectional nature, which prevented us from following longitudinal trajectories of TD children’s patterns of gesture comprehension and production. Even though our study provided some evidence for similarities between production and comprehension in the types of gestures and gesture + speech combinations children produced at each age group, there is still need for future studies that examine the link between these two processes in a longitudinal sample of TD children. Another limitation is the relatively modest sample size for each age group, which might have accounted for the lack of a significant age difference in overall gesture production—even if the older children (age 4) tended to produce a greater number of gestures than their younger peers.

Importantly, our findings showed a temporal association in the production and comprehension of gesture—a pattern that has been shown not to be true for speech development. We know from earlier work that comprehension and production of speech show marked temporal dissociations, with children showing earlier and better comprehension than production abilities at the early stages of language development (e.g., Bornstein & Hendricks, 2012; Fenson et al., 1994). Some authors even suggest that comprehension and production are “dissociated” psycholinguistic processes that draw on different skill sets (Bates et al., 1993; Dale & Goodman, 2005; Fenson et al., 1994). This was a pattern that was *not* evident in our study, where production and comprehension of gesture showed largely similar developmental trajectories. One reason for the difference could be that gesture expresses meaning through embodied action, and as such might not place the kinds of sensory demands words place on speech production. This possibility aligns with embodied cognition approaches that stress the active role children’s sensorimotor experiences play in building perception–action couplings in cognitive and language development (Hockema & Smith, 2009; Smith & Gasser, 2005). Thus, producing gestures might not only be similar to observing or understanding gesture in the extent of the cognitive effort involved, but be even necessary for young children to establish the mapping between gesture forms and their meanings (Rizzolatti & Arbib, 1998).

One question that remains is whether the close link between gesture and speech production in children’s achievement of milestones in earlier research might also be evident in the comprehension of gesture, and subsequently speech. Children identify a referent in gesture approximately three months before they produce word for the same referent (Iverson & Goldin-Meadow, 2005); similarly children convey different types of semantic relations in gesture + speech before expressing such relations entirely in speech four months later (Özçalışkan & Goldin-Meadow, 2005b, 2010). However, we do not yet know whether comprehension of a referent in gesture or a semantic relation across gesture + speech precedes and predicts its comprehension in speech by words and sentences, respectively. Future longitudinal studies that examine changes in gesture *and* speech comprehension with the same group of children are needed to shed light on the developmental trajectories associated with comprehension of gesture and speech.

In summary, our findings on the gesture production and comprehension of children between ages 2–4 showed that types of gesture and gesture + speech combinations followed largely similar trajectories in both production and comprehension. Thus, the temporal association between gesture comprehension and production appeared to be a robust aspect of communicative development, emerging early and remaining relatively stable in the early years. But is the alignment between comprehension and production of gesture also evident in a communicative system where production of gesture is negatively affected. We pursued this possibility in study 2, focusing on young children with ASD, who experience difficulties in gesture production (Mundy et al., 1986; Özçalışkan et al., 2018; Wetherby, 1986).

## Study 2

### Production and Comprehension of Gesture in Children with ASD

Children with ASD produce fewer gestures and begin producing gestures later than TD children (e.g., Choi et al., 2020; Colgan et al., 2006; Mishra et al., 2021; Mitchell et al., 2006; Mundy et al., 1986; Özçalışkan et al., 2016, 2017; Rapin, 1996). Some studies further suggest that children with ASD show atypical patterns in gesture production: for example, they might use parents instrumentally (e.g., place parent’s hand on a toy to make the parent activate the toy; Mastrogiuseppe et al., 2015) or use gestures to serve an instrumental function to receive objects (e.g., extend palm to request an object; Camaioni et al., 1997; Mundy et al., 1986). Importantly, this difference in gesture production is not evident in gesture comprehension, with verbal children with ASD showing levels of comprehension comparable to TD children (Dimitrova et al., 2017). There is, however, no work that has yet examined patterns of gesture production and comprehension in the same group of children with ASD. In study 2, we asked whether the comprehension and production of gesture in verbal children with ASD would show the same pattern of similarities and/or differences, and as compared to TD children.

Young children with ASD produce fewer gestures compared to TD children (Gulsrud et al., 2014; Mastrogiuseppe et al., 2015; Mundy et al., 1990)—even when they produce similar amounts of speech (Özçalışkan et al., 2016). At the same time, however, children with ASD produce similar types of gestures as TD children. A study that compared the production of gesture in young children with ASD and TD children found that both groups of children produced three gestures types—deictic, conventional and iconic—at similar distributional frequencies, with deictic gestures followed by conventional along with a few iconic gestures (Özçalışkan et al., 2016, 2018). Comprehension of gesture, on the other hand, presents mixed patterns. Some studies highlighted difficulties young children with ASD show in gesture comprehension, particularly for deictic gestures (Camaioni et al., 1997; Mundy et al., 1986), while others suggested otherwise, showing very similar patterns in children’s comprehension of different gesture types in both groups (Dimitrova et al., 2017).

Young children with ASD produce fewer but similar types of gesture + speech combinations compared to TD children, using gesture either to reinforce or supplement speech (Baumann et al., 2019; Choi et al., 2020; Özçalışkan et al., 2018). At the same time, unlike their TD peers, children with ASD produce fewer supplementary—but *not* reinforcing—gesture + speech combinations (Özçalışkan et al., 2018), suggesting that gesture might serve a different function in relation to speech for these children. Comprehension of gesture + speech also presents mixed findings: studies with adolescents with ASD show greater difficulties with supplementary gesture + speech combinations than language-comparable TD children (Hubbard et al., 2012; Silverman et al., 2010), while younger children with ASD show no evidence of a difference in their comprehension of gesture + speech combinations (Dimitrova et al., 2017) compared to TD children of similar language ability.

### The Current Study

Most of the earlier work focused on either production or comprehension of gesture in young children with ASD, leaving the parallel changes in production and comprehension of gesture unstudied. Research on gesture comprehension in children with ASD also remains very scarce, with mixed findings when compared to TD children. Thus, it is unknown whether difficulties in gesture production influence the alignment of patterns between gesture production and comprehension. We asked whether children with ASD would show a similar pattern in their production and comprehension of different types of gestures and gesture + speech combinations—thus mirroring the patterns observed in TD children. We predicted that difficulties in gesture comprehension would mirror difficulties in gesture production, based both on the relative weaknesses and delays that children with ASD show in gesture production (Colgan et al., 2006; Mitchell et al., 2006; Mundy et al., 1986; Özçalışkan et al., 2016, 2017; Rapin, 1996) and on the similarities we observed in patterns of comprehension and production for the types of gestures and gesture + speech combinations among TD children in Study 1.

## Method

### Participants

The sample consisted of 27 children with ASD (*M*_age_ = 5;8, range = 2;7–12;2, 20 boys),^4^ along with the 41 TD children (*M*_age_ = 3;5, range = 2;2–5;0, 17 boys) who formed the sample of study 1. As part of the inclusion criteria for the newly recruited ASD group, all children with ASD had a diagnosis of ASD assessed by a licensed clinician, scored outside the cutoff for autism on the Social Responsiveness Scale (SRS, Constantino & Gruber, 2005; (cutoff = 59 + ; *M*_ASD_ = 75.04, *SD* = 11.66) and were learning English as their native language.

Given the lack of age effects in TD children’s production and comprehension of different types of gestures and gesture + speech combinations in Study 1, we collapsed the three groups of TD children into one, and recruited a group of children with ASD so that they would be comparable to the TD children (collapsed across ages) in both expressive (EVT: *M*_TD_ = 51.38, *SD* = 15.69 vs. *M*_ASD_ = 52.08, *SD* = 19.28; *t*(60) = − 0.157, *p* = 0.875) and receptive language abilities (PPVT-IV: *M*_TD_ = 49.32, *SD* = 13.92 vs. *M*_ASD_ = 49.85, *SD* = 20.97; *t*(65) = − 0.124, *p* = 0.902).^5^

All children with ASD had a formal diagnosis for autistic disorder or pervasive developmental disorder not otherwise specified according to the DSM-IV-R criteria (American Psychiatric Association, 2013)—the most recent edition of DSM that was available during the clinical evaluation of participants for ASD. For all but two of the children, diagnoses were confirmed by documentation of a clinical evaluation by a licensed clinical psychologist using the Autism Diagnostic Interview-Revised (Rutter et al., 2003).^6^

The racial and socio-economic backgrounds were similar in the ASD and TD groups. The children with ASD were African-American (40%), Caucasian (37%), or mixed (23%). The majority of the parents of children with ASD had college (40%) or post-graduate degrees (34%). All parents provided written consent prior to participation in the study.

### Procedure for Data Collection

We followed the same procedures outlined in study 1.

### Procedure for Data Transcription and Coding

*Gesture production* We followed the same procedure used in study 1 for speech transcription and gesture coding in the gesture production task; we also assessed reliability with the same coders. One coder coded the entire data set for the autism sample and a second coder independently coded a randomly selected 15% of the data set. Intercoder agreement was 79% for identification of gestures, 99% for coding gesture into types, and 76% for coding gesture + speech into types.

*Gesture comprehension* We followed the same coding procedure in study 1 and assessed scoring reliability with the same coders. One coder scored all responses using video records. A second coder, blind to study hypotheses and child age, scored a randomly selected 20% of the responses in each age group. The agreement between coders was 98%.

### Scoring and Analysis

We used the same procedure for scoring and analysis for overall gesture production and comprehension as in study 1. We examined differences in language abilities, in overall production and comprehension of gesture between TD children and children with ASD using t-tests for independent samples. Gesture rates for either production or comprehension showed group differences (see Table 3). We, therefore, converted all raw frequencies for gesture types and gesture + speech combination types into proportions separately for production (see footnote 2) and comprehension (see footnote 3), transformed them using arcsine, and conducted all analyses on the arcsine-transformed proportions. We assessed differences in gesture production and gesture comprehension with separate mixed ANOVAs, with group (TD vs. ASD) as a between-subjects and either gesture type (deictic, conventional, iconic) or gesture + speech type (reinforcing gesture–speech combinations, supplementary gesture–speech combinations) as within-subject factors. All posthoc multiple comparisons were corrected, using Bonferroni.

**Table 3:** Mean number (standard deviation in parentheses) of gesture production (upper panel) and gesture comprehension (lower panel) by typically-developing (TD) children and children with autism spectrum disorder (ASD)

		TD (*n* = 41)	ASD (*n* = 27)
		Mean (*SD*)	Mean (*SD*)
*PRODUCTION*			
Gesture types	Overall	23.22 (14.35)	15.04 (12.01)
	Deictic	17.61 (12.52)	12.04 (10.10)
	Conventional	5.20 (6.01)	2.44 (4.96)
	Iconic	0.41 (0.77)	0.56 (0.93)
Gesture + speech combinations	Reinforcing	4.12(4.22)	3.40(6.28)
	Supplementary	5.51(8.72)	2.48(3.45)
*COMPREHENSION*			
Gesture types	Overall	26.12 (5.37)	24.78 (6.93)
	Deictic	9.85 (2.16)	8.89 (3.06)
	Conventional	7.63 (2.09)	7.74 (2.29)
	Iconic	8.63 (2.20)	8.15 (2.65)
Gesture + speech combinations	Reinforcing	7.07 (2.00)	6.59 (2.13)
	Supplementary	5.68 (1.55)	5.44(1.62)

## Results

**Fig. 6:**
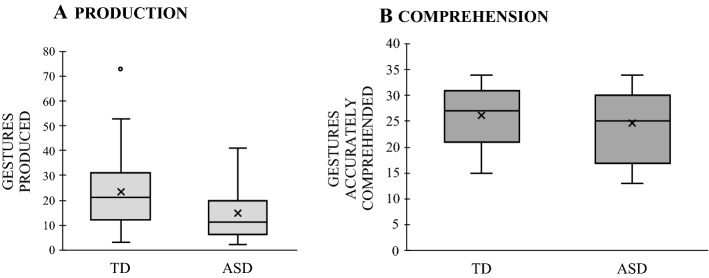
Mean number of gestures produced (**A**) and accurately comprehended (**B**) by typically-developing (TD) children and children with autism (ASD). The boxplot shows both the spread and the centers of the scores separately for production and comprehension. The measure of spread includes the interquartile range from 1st to 3rd quartiles (marked by the range of shaded columns) and score range (whiskers); the measure of centers includes the mean (x) and the median (marked by dark horizontal line within the shaded column)

We first examined group differences in children’s overall production and comprehension of gesture, and found differences in production, but *not* comprehension. As Fig. 6 shows, TD children produced significantly more gestures than children with ASD, *t*(66) = 2.449, *p* = 0.017 (6A), but remained comparable to their peers with ASD in gesture comprehension, *t*(66) = 0.898, *p* = 0.372 (6B).

**Fig. 7:**
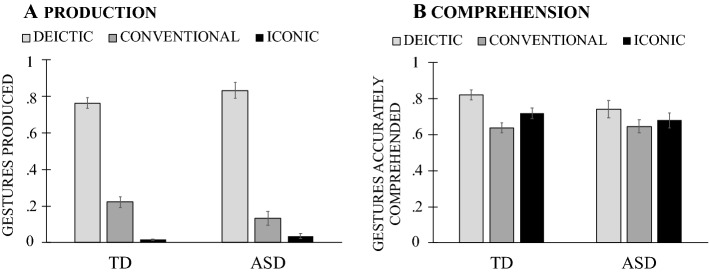
Mean production (**A**) and comprehension (**B**) of deictic, conventional and iconic gestures by children with typical development (TD) and with autism spectrum disorder (ASD)

Next, we asked whether children’s production and comprehension of different types of gestures and gesture + speech combinations remained similar in the two groups. Beginning with gesture types, children’s *production* showed no effect of group, *F*(1, 66) = 0.0001, *p* = 0.984, η^2^_p_ = 0.0001, but an effect of gesture type, *F*(2, 132) = 223.581, *p* = 0.0001, η^2^_p_ = 0.772, which interacted with group, *F*(2, 132) = 6.149, *p* = 0.003, η^2^_p_ = 0.085. Children produced a greater proportion of deictic than conventional (*p* < 0.001) and conventional than iconic gestures (*p* < 0.001; see Fig. 7A)—a pattern that was more pronounced for TD children. Pairwise comparisons for the interaction term revealed that TD children produced more deictic and conventional gestures than their peers with ASD (*p*_*s*_ < 0.05), but the two groups did not differ in terms of production of iconic gestures (*p* = 0.286). Gesture *comprehension* showed largely similar patterns, with no effect of group, *F*(1, 65) = 0.244, *p* = 0.623, η^2^_p_ = 0.004, but an effect of gesture type, *F*(2, 13) = 19.596, *p* = 0.0001, η^2^_p_ = 0.232, which did not interact with group, *F*(2, 130) = 1.389, *p* = 0.253, η^2^_p_ = 0.021; Fig. 7B). Children—across groups—showed better comprehension of deictic than iconic (*p*_*s*_ ≤ 0.01) and of iconic than conventional gestures (*p* = 0.017).

**Fig. 8:**
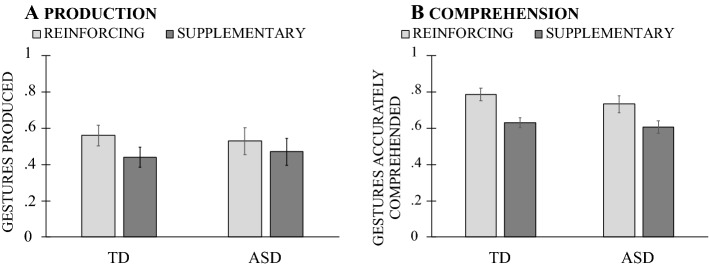
Mean production (**A**) and comprehension (**B**) of reinforcing and supplementary gestures-speech combinations by children with typical development (TD) and with autism spectrum disorder (ASD)

Turning next to gesture + speech combinations, children’s *production* showed no effect of group, *F*(1, 66) = 0.559, *p* = 0.457, η^2^_p_ = 0.008, no effect of gesture + speech type, *F*(1, 66) = 1.314, *p* = 0.256, η^2^_p_ = 0.020, and No Group × Gesture + Speech Type interaction, *F*(1, 66) = 0.559, *p* = 0.457, η^2^_p_ = 0.008 (Fig. 8A). The pattern was mostly similar for *comprehension*, with no effect of group, *F*(1, 65) = 0.627, *p* = 0.431, η^2^_p_ = 0.010, but an effect of combination type, *F*(1, 65) = 34.713, *p* = 0.0001, η^2^_p_ = 0.348, which did not interact with group, *F*(1, 65) = 0.066, *p* = 0.799, η^2^_p_ = 0.001. Children—across groups—showed greater comprehension of reinforcing than supplementary gestures (*p* < 0.001; see Fig. 8B).^7^

## Discussion

In study 2, we asked whether verbal children with ASD would follow the same patterns in their production and comprehension of gesture as TD children, and found evidence for it. Children in both groups showed better performance for deictic gestures than conventional and iconic gestures both in comprehension and production. The two groups also showed better performance for reinforcing than supplementary gesture + speech combinations—a difference that was only reliable for gesture comprehension. These results thus suggest that production and comprehension of different types of gestures and gesture + speech combinations largely follow similar patterns in children with ASD and with TD—even though children with ASD produced fewer gestures.

Our findings further support earlier work that showed lower gesture production in children with ASD compared to TD children (Choi et al., 2020; Mishra et al., 2021; Mundy et al., 1986; Özçalışkan et al., 2016). Importantly, however, this pattern was not evident in gesture comprehension: children with ASD were comparable to TD children in their ability to understand gestures. What might underlie this phenomenon? Research has attributed lower production of gesture among children with ASD to the difficulties they experience in establishing and sustaining joint attention (Adamson & Dimitrova, 2014; Adamson et al., 2009)—a difficulty that might be indicative of diagnosis-specific differences (Baron-Cohen, 1989; Dawson et al., 2004; Landa et al., 2007; Wetherby et al., 2004, 2007). Yet, the same children did not show such difficulties in comprehension of gesture, suggesting that gesture production and comprehension might place different demands on children with ASD. Production of gesture, different from comprehension, requires the child to more overtly engage in social interaction—a difference that might place greater communicative and cognitive demands on children with ASD, negatively affecting their performance.

At the same time, regardless of differences in overall amount of gesture production, children with ASD showed similar patterns in the gesture types they produced and comprehended—with better production and comprehension of deictic compared to conventional and iconic gestures. However, children with ASD did not differ from TD children in either their production or comprehension of deictic gestures—a finding that goes against our predictions and most of the earlier work on gesture production. One reason for this finding could be our study design: we assessed gesture production in a semi-structured parent–child play context with props known to elicit gestures (Baumann et al., 2019). To assess gesture comprehension, we used a structured task involving simple elicitation tasks with visible props in the immediate environment of the child. In contrast to our study, earlier work has examined production or comprehension of gesture indirectly, in either naturalistic (i.e., home videotapes; e.g., Baranek, 1999; Werner & Dawson, 2005) or semi-naturalistic contexts (Early Social Communication Scales: Mundy et al., 2003; Autism Diagnostic Observation Schedule: Lord et al., 2012), or by using indirect measures, such as parent report (MacArthur Communicative Development Inventories: Fenson et al., 2003). Importantly, these tools were not specifically designed to assess gesture comprehension or production, but were tools to screen autism symptoms. The difference in the findings thus might be attributable to the greater flexibility and ease of the elicitation methods in our study.

A second possible explanation for the lack of difference in deictic gesture production and comprehension might be the characteristics of our sample of children with ASD. Compared to earlier work that showed lower rates of deictic gesture production (Mundy et al., 1986; Özçalışkan et al., 2016, 2017) and comprehension (Camaioni et al., 1997; Mundy et al., 1986) in young children with ASD, our study included older children with ASD (*M*_age_ = 5;8, range = 2;7–12;2) with relatively strong verbal abilities—equivalent to that of a 4-year-old TD child. This raises the possibility that either age or verbal ability might help explain the discrepancy of our findings compared to earlier work. Importantly, in our ASD sample gesture comprehension was strongly correlated with receptive language (*r* = 0.803 *p* = 0.0001) but not with chronological age (*r* = 0.104, *p* = 0.605), suggesting that verbal ability might be a key contributor to gesture comprehension.

Following a pattern akin to gesture types, children with ASD showed similar patterns in the types of gesture + speech combinations that they produced and comprehended. More specifically, they showed lower comprehension and tended to show lower production of supplementary gesture + speech combinations—mirroring a pattern observed in TD children. One likely explanation for the similar patterns in the two groups could be the comparable verbal age: supplementary combinations that convey different pieces of information in each modality might have placed similar cognitive demands on both groups of children.

One potential limitation of our study is that all of the children with ASD in our sample were verbal, thus limiting the generalizability of our results to verbal children with ASD. Future studies that extend this work to younger and/or less verbal children with ASD can shed further light on the potential effect of language ability on patterns of gesture production and comprehension in young children’s early communications.

Taken together, our findings indicate similarities in patterns of comprehension and production in both children with ASD and with TD, suggesting that the communicative and cognitive mechanisms underlying production and comprehension of gesture might be similar in both groups of children.

### Conclusion

Gesture constitutes a robust feature of children’s early communication. Young children not only *produce gestures* to share information, but also *glean information* from the gestures of others to expand their understanding of the world. Here we asked whether the developmental trajectory children follow in production and comprehension of gesture remains similar in TD children, and if so, whether the close alignment between production and comprehension also extends to children with ASD, who show marked delays and difficulties in gesturing. Our results showed that children’s comprehension of gesture largely mirrored their production in both typical development and autism, suggesting that comprehension and production of gesture constitute closely integrated systems of communication across learners.

## Data Availability

Data is available upon request from the corresponding author.

## Footnotes

1 The original sample consisted of 49 TD children. Eight children were excluded due to developmental concerns (e.g., scoring above cutoff on the SRS: score > 59; Constantino & Gruber, 2005), premature birth or stay in a neonatal intensive care unit *n* = 6) or due to experimental error (*n* = 2).

2 We computed the proportions by dividing each gesture type (deictic, conventional, iconic) or gesture + speech type (reinforcing, supplementary) type a child produced by the total number of gestures or gesture + speech combinations produced by the child.

3 We computed the proportions by dividing each child’s comprehension score for each gesture type (e.g., deictic) or gesture + speech type (e.g., reinforcing) by the total number of items with the same gesture or gesture + speech type the child was tested on. For example, for comprehension of deictic gestures we divided the total number of deictic gestures a child comprehended by the total number of items with deictic gestures that the child was tested on.

4 The original sample included 48 children with ASD; however, we had to exclude 21 children because their score on the Expressive Language Test (EVT-2; Williams, 2007) was outside the range of expressive language abilities for the 41 TD children (i.e., they were nonverbal or already producing complex speech, *n* = 9), or they did not complete the gesture comprehension task (*n* = 9) or their data had too poor video quality to allow for gesture coding (*n* = 3).

5 We used EVT and PPVT standardized age equivalent scores instead of raw scores in making the two groups comparable because of the considerable variability in age range, particularly within the ASD group, following earlier work on children with Williams syndrome (Mervis & Klein-Tasman, 2004). The comparability of the groups was established with an independent t-test that statistically compares mean differences between the two groups.

6 For two of the children in the ASD group, we did not have access to a formal written documentation to further confirm parents’ report of the clinical diagnoses; both of these children however met the cutoff for autism in the SRS measure, scoring at 76 and above and thus indicating severe autism.

7 We tested whether there is a positive relation between children’s production and comprehension of gesture for different types of gestures and gesture + speech combinations. Our results showed no systematic correlations between production and comprehension for either TD children (Spearman’s rho = 0.141, *p* = 0.379) or children with ASD (Spearman’s rho = 0.100, *p* = 0.621).

## References

[CR1] Adamson LB, Dimitrova N, Brooks PJ, Kempe V (2014). Joint attention and language development. Encyclopedia of language development.

[CR2] Adamson LB, Bakeman R, Deckner DF, Romski M (2009). Joint engagement and the emergence of language in children with autism and down syndrome. Journal of Autism and Developmental Disorders.

[CR3] American Psychiatric Association. (2013). *Diagnostic and statistical manual of mental disorders* (5th Edn.). Washington, DC.

[CR4] Baranek GT (1999). Autism during infancy: A retrospective video analysis of sensory-motor and social behaviors at 9–12 months of age. Journal of Autism and Developmental Disorders.

[CR5] Baron-Cohen S (1989). Perceptual role taking and protodeclarative pointing in autism. British Journal of Developmental Psychology.

[CR6] Bates E (1976). Language and context: The acquisition of pragmatics.

[CR7] Bates E (1993). Comprehension and production in early language development: Comments on Savage-Rumbaugh et al. Monographs of the Society for Research in Child Development.

[CR8] Bates E, Benigni L, Bretherton I, Camaioni L, Volterra V (1979). The emergence of symbols: Cognition and communication in infancy.

[CR9] Bates E, Thal D, Whitesell K, Fenson L, Oakes L (1989). Integrating language and gesture in infancy. Developmental Psychology.

[CR10] Baumann S, Özçalışkan Ş, Adamson LB (2019). Do early school-aged children’s gestures reflect parental gesture input in autism and typical development?. Research in Autism Spectrum Disorders.

[CR11] Behne T, Liszkowski U, Carpenter M, Tomasello M (2012). Twelve-month-olds’ comprehension and production of pointing. British Journal of Developmental Psychology.

[CR12] Bekken, K. (1989). *Is there motherese in gesture?*. Unpublished doctoral dissertation. Chicago, IL: The University of Chicago.

[CR13] Bornstein MH, Hendricks C (2012). Basic language comprehension and production in> 100,000 young children from sixteen developing nations. Journal of Child Language.

[CR14] Butcher C, Goldin-Meadow S, McNeill D (2000). Gesture and the transition from one- to two-word speech: When hand and mouth come together. Language and gesture.

[CR15] Camaioni L, Perucchini P, Muratori F, Milone A (1997). Brief report: A longitudinal examination of the communicative gestures deficit in young children with autism. Journal of Autism and Developmental Disorders.

[CR16] Camaioni L, Perucchini P, Bellagamba F, Colonnesi C (2004). The role of declarative pointing in developing a theory of mind. Infancy.

[CR17] Capone NC, McGregor KK (2004). Gesture development: A review for clinical and research practices. Journal of Speech, Language, and Hearing Research.

[CR18] Carpenter M, Nagell K, Tomasello M, Butterworth G, Moore C (1998). Social cognition, joint attention and communicative competence from 9 to 15 months of age. Monographs of the Society for Research in Child Development.

[CR19] Choi B, Shah P, Rowe ML, Nelson CA, Tager-Flusberg H (2020). Gesture development, caregiver responsiveness, and language and diagnostic outcomes in infants at high and low risk for autism. Journal of Autism and Developmental Disorders.

[CR20] Colgan S, Lanter E, McComish C, Watson L, Crais E, Baranek G (2006). Analysis of social interaction gestures in infants with autism. Child Neuropsychology.

[CR21] Colonnesi C, Rieffe C, Koops W, Perucchini P (2008). Precursors of theory of mind. A longitudinal study. British Journal of Developmental Psychology.

[CR22] Colonnesi C, Stams GJJM, Koster I, Noom MJ (2010). The relationship between pointing gesture and language: A meta-analysis. Developmental Review.

[CR23] Constantino JN, Gruber CP (2005). Social Responsiveness Scale (SRS).

[CR24] Dale P, Goodman J, Tomasello M, Slobin DI (2005). Commonality and individual differences in vocabulary growth. Beyond nature–nurture. Essays in honor of Elizabeth Bates.

[CR25] Dawson G, Toth K, Abbott R, Osterling J, Munson J, Estes A, Liaw J (2004). Early social attention impairments in autism: Social orienting, joint attention, and attention to distress. Developmental Psychology.

[CR26] Demir OE, Levine S, Goldin-Meadow S (2015). A tale of two hands: Development of narrative structure in children’s speech and gesture. Journal of Child Language.

[CR27] Dimitrova N, Özçalıskan S, Adamson LB (2017). Do verbal children with autism comprehend gesture as readily as typically developing children?. Journal of Autism and Developmental Disorders.

[CR28] Dunn L, Dunn D (2007). PPVT-4, peabody picture vocabulary test manual.

[CR29] Fenson L, Dale PS, Reznick JS, Bates E, Thal DJ, Pethick SJ (1994). Variability in early communicative development. Monographs of the Society for Research in Child Development.

[CR30] Fenson L, Dale P, Reznick J, Thal D, Bates E, Hartung J, Pethick S, Reilly J (2003). MacArthur communicative development inventories: User’s guide and technical manual.

[CR31] Goldin-Meadow S (2014). Widening the lens: What the manual modality reveals about language, learning and cognition. Philosophical Transactions of the Royal Society.

[CR32] Goodwyn S, Acredolo L, Iverson JM, Goldin-Meadow S (1998). Encouraging symbolic gestures: A new perspective on the relationship between gesture and speech. The nature and functions of gesture in children’s communication.

[CR33] Greenfield P, Smith J (1976). The structure of communication in early language development.

[CR34] Gulsrud AC, Hellemann GS, Freeman SFN, Kasari C (2014). Two to ten years: Developmental trajectories of joint attention in children with ASD who received targeted social communication interventions. Autism Research.

[CR35] Hockema SA, Smith LB (2009). Learning your language, outside-in and inside-out. Linguistics.

[CR36] Hodges LE, Özçalışkan Ş, Williamson R (2018). Type of iconicity influences children’s comprehension of gesture. Journal of Experimental Child Psychology.

[CR37] Hoff, E. (2012). *Transcription manual.* The Language Development Lab of Florida Atlantic University.

[CR38] Hubbard AL, McNealy K, Scott-Van Zeeland AA, Callan DE, Bookheimer SY, Dapretto M (2012). Altered integration of speech and gesture in children with autism spectrum disorders. Brain and Behavior.

[CR39] Iverson JM, Capirci O, Caselli MC (1994). From communication to language in two modalities. Cognitive Development.

[CR40] Iverson JM, Capirci O, Longobardi E, Caselli MC (1999). Gesturing in mother-child interactions. Cognitive Development.

[CR41] Iverson JM, Capirci O, Volterra V, Goldin-Meadow S (2008). Learning to talk in a gesture-rich world: Early communication of Italian versus American children. First Language.

[CR42] Iverson JM, Goldin-Meadow S (2005). Gesture paves the way for language development. Psychological Science.

[CR43] Landa RJ, Holman KC, Garrett-Mayer E (2007). Social and communication development in toddlers with early and later diagnosis of autism spectrum disorders. Archives of General Psychiatry.

[CR44] Limia VD, Özçalışkan Ş, Hoff E (2019). Do parents provide a helping hand to vocabulary development in bilingual children?. Journal of Child Language.

[CR45] Liszkowski U, Carpenter M, Striano T, Tomasello M (2006). 12-and 18-month-olds point to provide information for others. Journal of Cognition and Development.

[CR46] Lord, C., Rutter, M., DiLavore, P. C., Risi, S., Gotham, K., & Bishop, S. L. (2012). *Autism diagnostic observation schedule, (ADOS-2) manual (Part 1): Modules 1–4* (2nd Edn.). Western Psychological Services.

[CR47] Mastrogiuseppe M, Capirci O, Cuva S, Venuti P (2015). Gestural communication in children with autism spectrum disorders during mother–child interaction. Autism.

[CR48] Mervis CB, Klein-Tasman BP (2004). Methodological issues in group-matching designs: Alpha levels for control variable comparisons and measurement characteristics of control and target variables: Research methodology-matching. Journal of Autism and Developmental Disorders.

[CR49] Mishra A, Ceballos V, Himmelwright K, McCabe S, Scott L (2021). Gesture production in toddlers with Autism Spectrum Disorder. Journal of Autism and Developmental Disorders.

[CR50] Mitchell S, Brian J, Zwaigenbaum L, Roberts W, Szatmari P, Smith I (2006). Early language and communication development of infants later diagnosed with autism spectrum disorder. Journal of Developmental Behavioral Pediatrics.

[CR51] Morford M, Goldin-Meadow S (1992). Comprehension and production of gesture in combination with speech in one-word speakers. Journal of Child Language.

[CR52] Mundy P, Sigman MD, Ungerer J, Sherman T (1986). Defining the social deficits of autism: The contribution of non-verbal communication measures. Journal of Child Psychology and Psychiatry and Allied Disciplines.

[CR53] Mundy P, Sigman M, Kasari C (1990). A longitudinal study of joint attention and language development in autistic children. Journal of Autism and Developmental Disorders.

[CR54] Mundy P, Delgado C, Block J, Venezia M, Hogan A, Seibert J (2003). A manual for the Abridged Early Social Communication Scales (ESCS).

[CR55] Namy L (2008). Recognition of iconicity doesn’t come for free. Developmental Science.

[CR56] Namy L, Campbell A, Tomasello M (2004). Developmental change in the role of iconicity in symbol learning. Journal of Cognition & Development.

[CR57] O’Neill M, Bard KA, Linnell M, Fluck M (2005). Maternal gestures with 20-month-old infants in two contexts. Developmental Science.

[CR58] Özçalışkan Ş (2007). Metaphors we ‘move by’: Children’s developing understanding of metaphorical motion in typologically distinct languages. Metaphor and Symbol.

[CR59] Özçalışkan Ş, Goldin-Meadow S (2005). Do parents lead their children by the hand?. Journal of Child Language.

[CR60] Özçalıskan S, Dimitrova N (2013). How gesture input provides a helping hand to language development. Seminars in Speech and Language.

[CR61] Özçalışkan Ş, Goldin-Meadow S (2005). Gesture is at the cutting edge of early language development. Cognition.

[CR62] Özçalışkan Ş, Goldin-Meadow S (2009). When gesture-speech combinations do and do not index linguistic change. Language and Cognitive Processes.

[CR63] Özçalışkan Ş, Goldin-Meadow S (2010). Sex differences in language first appear in gesture. Developmental Science.

[CR64] Özçalışkan Ş, Goldin-Meadow S, Stam G, Ishino M (2011). Is there an iconic gesture spurt at 26 months?. Integrating gestures: The interdisciplinary nature of gesture.

[CR65] Özçalışkan Ş, Hodges L, Aydin C, Goksun T, Kuntay A, Tahiroglu D (2016). Jestlerin Çocukların Dilsel ve Bilişsel Gelişimindeki Rolü (Role of gesture in cognitive and linguistic development). Aklın Çocuk Hali: Zihin Gelişimi Araştırmaları (Studies on Cognitive Development).

[CR66] Özçalışkan Ş, Goldin-Meadow S, Gentner D, Mylander C (2009). Does language about similarity foster similarity comparisons in children?. Cognition.

[CR67] Özçalışkan Ş, Gentner D, Goldin-Meadow S (2014). Do iconic gestures pave the way for children’s early verbs?. Applied Psycholinguistics.

[CR68] Özçalışkan Ş, Adamson LB, Dimitrova N (2016). Early deictic but not other gestures predict later vocabulary in both typical development and autism. Autism.

[CR69] Özçalışkan Ş, Adamson LB, Dimitrova N, Baumann S (2017). Early gesture provides a helping hand to spoken vocabulary development for children with autism, Down syndrome and typical development. Journal of Cognition and Development.

[CR70] Özçalışkan Ş, Adamson LB, Dimitrova N, Baumann S (2018). Do parents model gestures differently when children’s gestures differ?. Journal of Autism and Developmental Disorders.

[CR71] Öztürk S, Pınar E, Ketrez N, Özçalışkan Ş (2021). Effect of sex and dyad type on speech and gesture development of singleton and twin children. Journal of Child Language.

[CR72] Pınar E, Öztürk S, Ketrez N, Özçalışkan Ş (2021). Effect of child sex and sibling composition on parental verbal and nonverbal input. Journal of Nonverbal Behavior Behavior.

[CR73] Ramos-Cabo S, Vulchanov V, Vulchanova M (2019). Gesture and language trajectories in early development: An overview from the autism spectrum disorder perspective. Frontiers in Psychology.

[CR74] Rapin I (1996). Preschool children with inadequate communication: Developmental language disorder, autism, low IQ.

[CR75] Rizzolatti G, Arbib MA (1998). Language within our grasp. Trends in Neurosciences.

[CR76] Rutter M, LeCouteur A, Lord C (2003). Autism diagnostic interview-revised.

[CR77] Scaife M, Bruner J (1975). The capacity for joint visual attention in the infant. Nature.

[CR78] Shore C, Bates E, Bretherton I, Beeghly M, O’Connell B, Volterra V, Erting C (1990). Vocal and gestural symbols: Similarities and differences from 13 to 28 months. From gesture to language in hearing and deaf children.

[CR79] Silverman L, Bennetto L, Campana E, Tanenhaus MK (2010). Speech-and-gesture integration in high-functioning autism. Cognition.

[CR80] Smith L, Gasser M (2005). The development of embodied cognition: Six lessons from babies. Artificial Life.

[CR81] Stanfield C, Williamson R, Özçalışkan Ş (2014). How early do children understand gesture–speech combinations with iconic gestures?. Journal of Child Language.

[CR82] Stites LJ, Özçalışkan Ş (2017). Who did what to whom? Children track story referents first in gesture. Journal of Psycholinguistic Research.

[CR83] Werner E, Dawson G (2005). Validation of the phenomenon of autistic regression using home videotapes. Archives of General Psychiatry.

[CR84] Wetherby A (1986). Ontogeny of communication functions in autism. Journal of Autism and Developmental Disorders.

[CR85] Wetherby AM, Woods J, Allen L, Cleary J, Dickinson H, Lord C (2004). Early indicators of autism spectrum disorders in the second year of life. Journal of Autism and Developmental Disorders.

[CR86] Wetherby AM, Watt N, Morgan L, Shumway S (2007). Social communication profiles of children with autism spectrum disorders late in the second year of life. Journal of Autism and Developmental Disorders.

[CR87] Williams KT (2007). Expressive vocabulary test—second edition (EVT–2). Journal of the American Academy of Child & Adolescent Psychiatry.

[CR88] Woodward AL, Guajardo JJ (2002). Infants’ understanding of the point gesture as an object-directed action. Cognitive Development.

